# Big Science vs. Little Science: How Scientific Impact Scales with Funding

**DOI:** 10.1371/journal.pone.0065263

**Published:** 2013-06-19

**Authors:** Jean-Michel Fortin, David J. Currie

**Affiliations:** Ottawa-Carleton Institute of Biology, University of Ottawa, Ottawa, Ontario, Canada; Université de Montréal, Canada

## Abstract

Agencies that fund scientific research must choose: is it more effective to give large grants to a few elite researchers, or small grants to many researchers? Large grants would be more effective only if scientific impact increases as an accelerating function of grant size. Here, we examine the scientific impact of individual university-based researchers in three disciplines funded by the Natural Sciences and Engineering Research Council of Canada (NSERC). We considered four indices of scientific impact: numbers of articles published, numbers of citations to those articles, the most cited article, and the number of highly cited articles, each measured over a four-year period. We related these to the amount of NSERC funding received. Impact is positively, but only weakly, related to funding. Researchers who received additional funds from a second federal granting council, the Canadian Institutes for Health Research, were not more productive than those who received only NSERC funding. Impact was generally a decelerating function of funding. Impact per dollar was therefore lower for large grant-holders. This is inconsistent with the hypothesis that larger grants lead to larger discoveries. Further, the impact of researchers who received increases in funding did not predictably increase. We conclude that scientific impact (as reflected by publications) is only weakly limited by funding. We suggest that funding strategies that target diversity, rather than “excellence”, are likely to prove to be more productive.

## Introduction

Consider a publically-funded research granting council with a fixed amount of money at its disposal. Is it more effective to give small grants to many researchers (what we shall call the “many small” strategy), or large grants to a chosen few (“few big”)? Can a granting agency manage scientific output/impact by rewarding researchers with larger grants? Do larger grants foster “excellence”?

Historically, the Natural Sciences and Engineering Research Council of Canada (NSERC) funded most scientists in Canadian universities (62% in 2012; [1]. To keep success rates high, NSERC typically awarded relatively small grants [2], [3]. In contrast, the U.S. National Science Foundation (NSF) awards much larger grants, with much lower success rates (23% in 2010; [4]. However, NSERC is moving away from the “many small” model [2], [5], [6] with the stated goal of “…[making] it possible for high-performing researchers to quickly increase their grant levels based on superior scientific merit” [7]. But does greater funding for high performers lead to greater scientific impact, versus funding more researchers?

The answer depends upon the goals of the funding program. The goal could be to maximize major discoveries: prize-winning work published in top journals. This strategy is usually presented as emphasizing excellence [7]; it can also be criticized as “photo-opportunity science” (G. McBean, quoted in [5]: a quest for national bragging rights). If the probability of major discoveries increases with funding, then the optimal strategy may be to concentrate resources on likely prize-winners.

Alternatively, the goal of the funding program could be to maximize the summed impact of a scientific community (e.g., university researchers). Optimal allocation of research funds in this case depends upon the shape of the relationship between the scientific impact of individual grantees and their funding. Suppose that scientific impact (*I*) of individual grantees over a given period varies as a power function of funding (*F*):
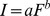
Alternatively :
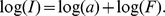


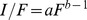
where *a* and *b* are empirical constants. There may be diminishing returns in research: generous budgets may be used less efficiently than tight budgets, such that increasing a researcher's funding by X% increases scientific output, but by less than X%. In this case, individual impact (*I*) would be a decelerating function of funding, implying that 0<*b*<1. Impact per dollar (*I/F*) would decrease with grant size *F* (Figure S1 in File S1). In this case, many small grants should yield greater total impact than a few big grants.

In contrast, larger grants may permit larger research groups of students, technicians, etc., whose interactions catalyse overall productivity. In this case, the impact of the individual grant holder is an accelerating function of funding, implying that *b*>1. Large grants should yield greater impact than dividing the same funds among multiple grants. When b = 1, impact per dollar is independent of grant size (Figure S1 in File S1).

The present study asks: how does scientific impact vary with individual researchers' grant sizes? Do well-funded researchers yield more “bang for the buck”? In operational terms, how strong is the relationship between *log(I)* and *log(F)*, and what is its slope b? Further, how does a researcher's scientific impact change in response to increased grant size? The implication, of course, is the broader question: how should granting agencies allocate their funds?

## Materials and Methods

It is convenient to address these questions using NSERC funding data, as NSERC is the principle, or sole, source of operating funds for many university researchers in basic sciences in Canada [2]. We used the Award Search Engine on the NSERC website [8] to obtain the amounts of research operating grants (i.e., funds for supplies, student salaries, field work, etc.) awarded to individual researchers in the Discovery Grants program of NSERC for the 2002 competition year in three disciplines: Integrative Animal Biology (Bio, n = 126), Inorganic & Organic Chemistry (Chem., n = 109) and Evolution & Ecology (Eco., n = 139). We calculated the total amount awarded by NSERC to the 2002 cohort over two four-year periods (2002–2003 to 2005–2006 and 2006–2007 to 2009–2010). For grantees who received awards in 2002 for less than four years, we added in subsequent grants received from NSERC during the study period, if any. We also tallied grant support received in the subsequent four years (2006–2007 to 2009–2010).

Some researchers obtain funding from more than one federal granting council. Most notably, researchers with health-related research (including many in Animal Biology and in Chemistry) may receive operating funds from both NSERC and the Canadian Institutes of Health Research (CIHR). We extracted information on CIHR grants from the Canadian Research Information System [9]. Since CIHR often awards team grants, we analyzed the data using the assumption that the lead investigators share 75% of the grant, and co-investigators equally share the rest. Other assumptions about how grants were shared among investigators did not qualitatively influence our results.

We also noted which researchers had received funding from the Canadian Foundation for Innovation (CFI). CFI provides large grants for infrastructure and major equipment purchases, but not for operating expenses. CFI funding data are presented on their website [10]. We also noted which researchers had concurrent funding from the Fonds Québécois de Recherche – Nature et Technologies (FQRNT), using their website [11]. FQRNT grants are similar to NSERC grants, but they are only available to researchers in the province of Québec. We treated CFI and FQRNT grants as categorical variables (funding received, or not), since large portions of CFI may go into major infrastructure such as buildings, and FQRNT grants are difficult to partition among participants. Essentially, we asked: did researchers who received CFI and/or FQRNT funding as well as NSERC funding have greater scientific impact than researchers with only NSERC?

We used four measures of scientific impact of individual grantees. First, we first tallied the number of peer-reviewed publications published from 2003 to 2006, and from 2007–2010 (one year off-set from the grant periods above) as listed on the Web of Science with the grantee as an author. We did not (nor does NSERC) try to distinguish publications funded by NSERC from research funded from other sources. Second, we determined the number of articles citing these publications on the Web of Science (excluding self-citations) as of March 2012. Third, since producing a single prominent study might be more highly valued by NSERC than many small studies, we noted the number of citations of the grantee's most highly cited paper during the same period (2003–2006). Finally, we also noted the number of papers published in 2003–2006 by the grantee that had received more than a threshold number of citations, representing the approximately 5% of most highly cited papers in the discipline during that period. Campbell et al. [12] discuss caveats of the use of bibliometric measures of scientific productivity such as the ones we used.

Because both funding and impact measures were strongly right-skewed, we log-transformed all variables (Figure S2 in File S1). Statistical analyses were carried out using R version 2.15.1 [13].

## Results and Discussion

**Figure 1:**
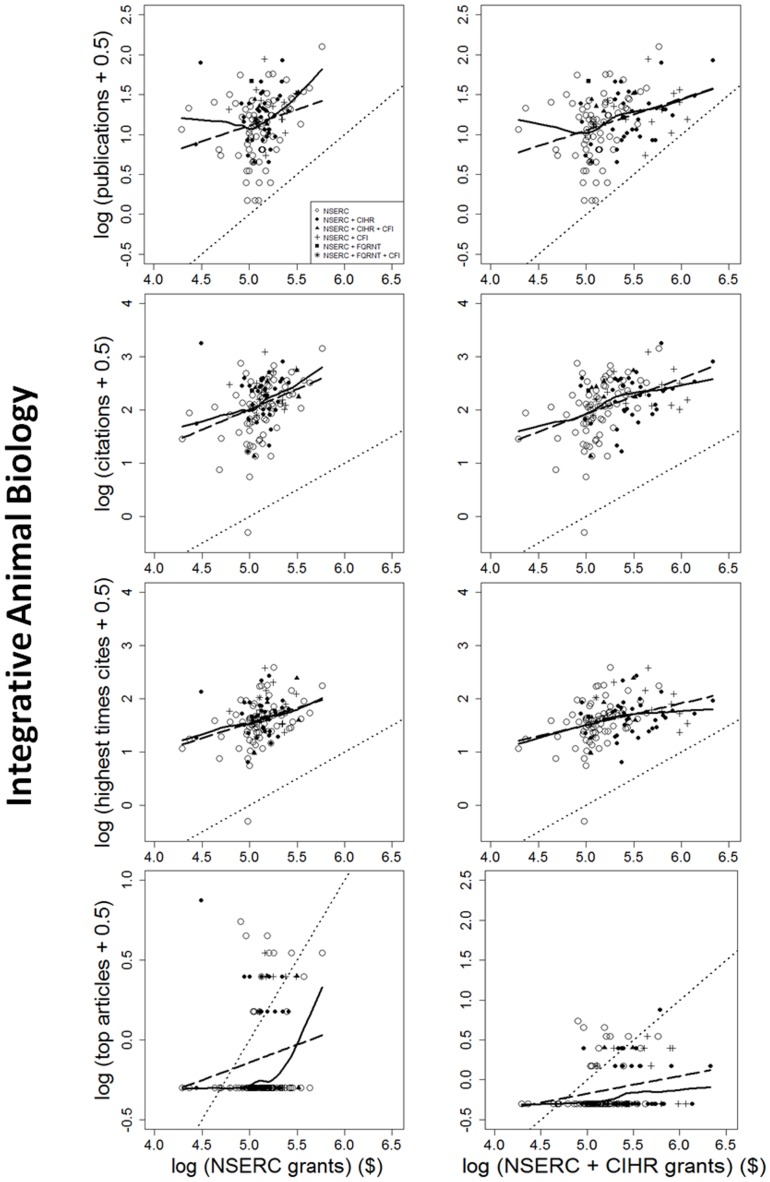
Four measures of the scientific impact of individual researchers from 2003 to 2007, expressed as functions of the logarithm of each researcher's NSERC Discovery grant (i.e. operating grant received from the Natural Sciences and Engineering Research Council of Canada) (left column), or the NSERC grant plus the researcher's grant from the Canadian Institutes for Health Research (CIHR), if any, in 2002 to 2006. The measures of scientific impact are: the total numbers of papers published, numbers of citations to those publications by 2012, the number of citations received by the most highly cited paper, and the number of very highly cited papers. The solid lines represent LOWESS (model-free) fits to the data. Dashed lines show linear regression fits to the log-transformed data. Dotted lines show a slope of 1.0. Symbols distinguish researchers who held only an NSERC grant, versus those who also held a grant from CIHR, CFI (the Canadian Foundation for Innovation) and/or the Fonds Québécois de Recherche – Nature et Technologies (FQRNT). Results are shown for scientists funded by the NSERC grant selection committee in Integrative Animal Biology. In all cases, individual impact increases with funding with a slope ≤1.0. Thus, impact is a decelerating function of grant size. Researchers who held grants other than NSERC are not significantly more productive than those who did not.

**Figure 2:**
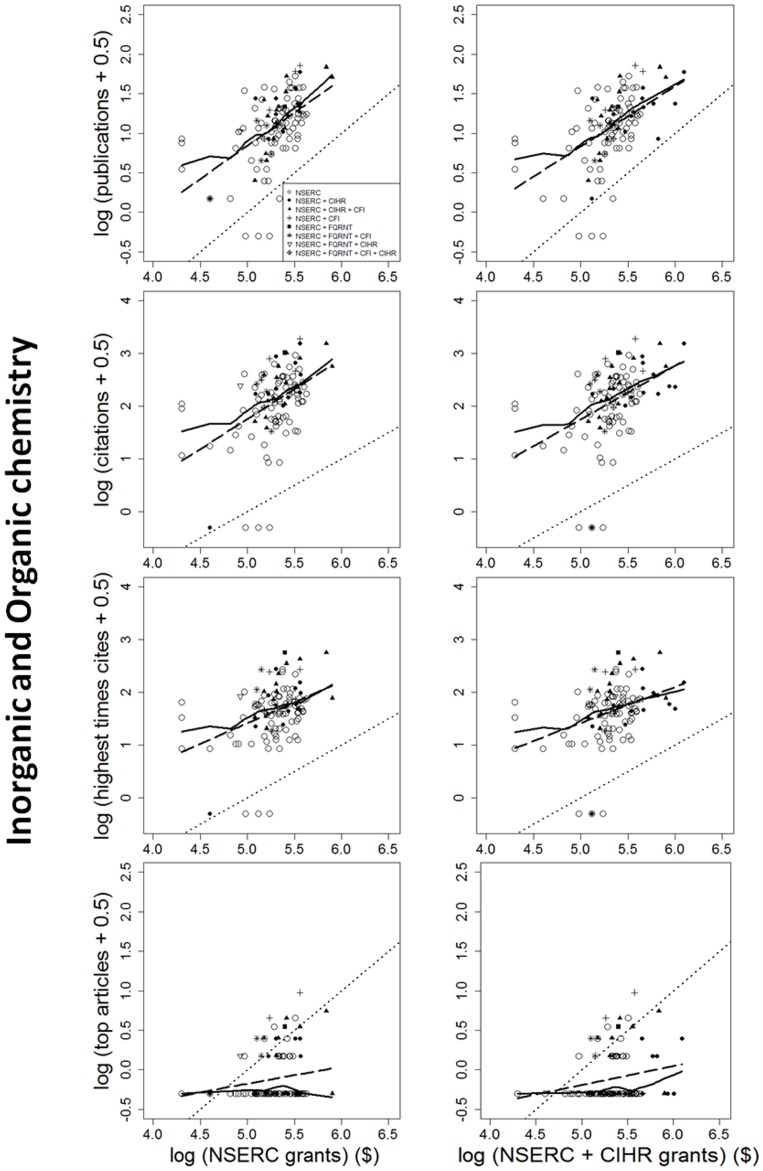
Four measures of the scientific impact of individual researchers from 2003 to 2007, expressed as functions of the logarithm of each researcher's NSERC Discovery grant. Details are the same as in Figure 1, except that results shown here are for scientists funded by the NSERC grant selection committee in Organic and Inorganic Chemistry. Again, individual impact increases with funding with a slope ≤1.0, and researchers who held grants other than NSERC are not significantly more productive than those who did not.

**Figure 3:**
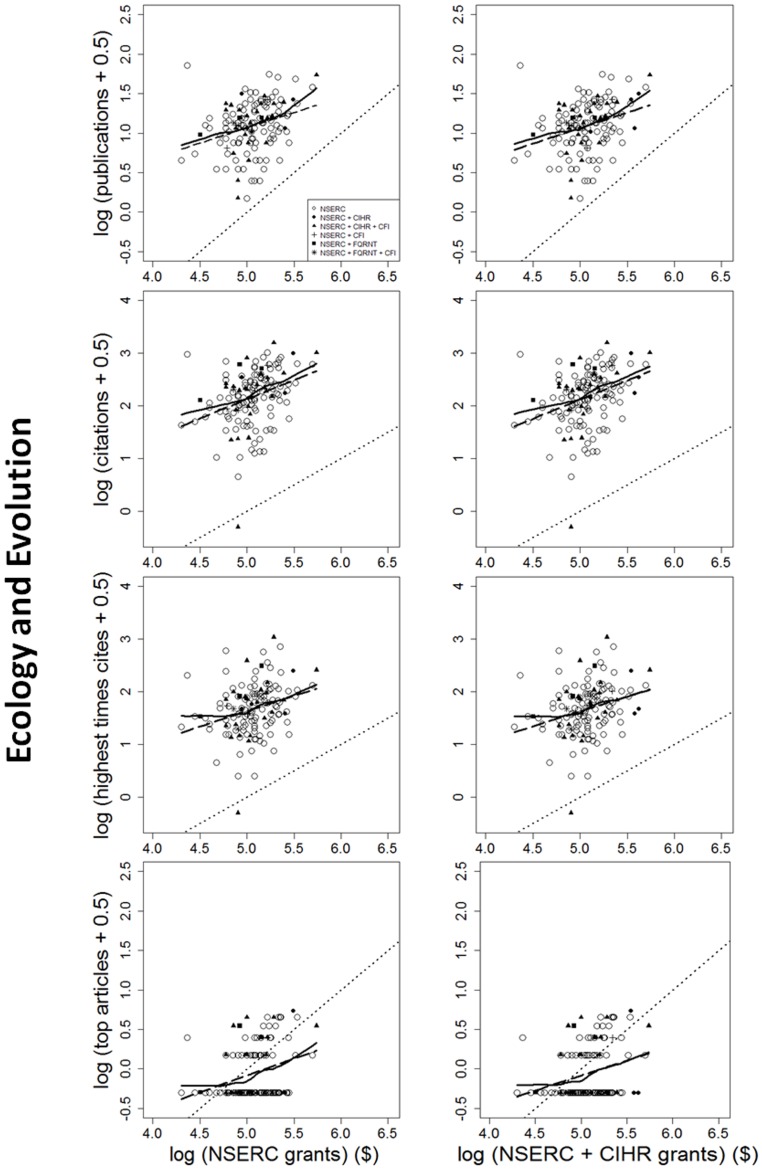
Four measures of the scientific impact of individual researchers from 2003 to 2007, expressed as functions of the logarithm of each researcher's NSERC Discovery grant. Details are the same as in Figures 1 and 2, except that results shown here are for scientists funded by the NSERC grant selection committee in Ecology and Evolution. Once again, individual impact increases with funding with a slope ≤1.0, and researchers who held grants other than NSERC are not significantly more productive than those who did not.

The impact of individual researchers increased with the amount of NSERC funding they had received. This was true for all the metrics of impact investigated (Figures 1, 2, 3). Berg [14] observed that the productivity of researchers funded by the U.S. National Institutes of Health (NIH) plateaued with grants larger than $700,000 per annum. Our data show no plateaus, but NSERC grants are all far below the NIH plateau. More surprising is that the impact-funding relationships are quite weak (Table 1), accounting for, at most, 28% of the among-researcher variation in impact. There may be a threshold of ∼$16,000–$25,000 per year below which impact remains constant; however, there is insufficient statistical power to support changes in slope. Results are very similar for the two funding periods; only the earlier period is shown. In sum, greater productivity is not strongly related to greater funding.

**Table 1:** Slopes (b) and their standard errors (SE) from linear models relating the logarithm of total scientific impact to the logarithm of grant support received from Individual Discovery grants from the Natural Sciences and Engineering Research Council of Canada (NSERC) in 2002–2006.

Impact measure	Committee	B.P.	b	SE	p_b = 0_	p_b = 1_	r^2^
a) Publications	Bio	-	0.40	(0.14)	**	***	0.06
	Bio	5.0	0.98	(0.21)	***	ns	0.18
	Chem.	-	0.84	(0.13)	***	ns	0.28
	Chem.	4.8	1.12	(0.18)	***	ns	0.27
	Eco	-	0.39	(0.11)	***	***	0.08
b) Citations	Bio	-	0.76	(0.19)	***	ns	0.11
	Chem.	-	1.14	(0.21)	***	ns	0.21
	Eco	-	0.73	(0.18)	***	ns	0.11
c) Highest times	Bio	-	0.57	(0.15)	***	**	0.11
cited	Chem.	-	0.79	(0.18)	***	ns	0.15
	Eco	-	0.58	(0.18)	**	**	0.07
d) Number of	Bio	-	0.22	(0.12)	#	***	0.03
high-impact	Chem.	-	0.22	(0.11)	#	***	0.03
articles	Eco	-	0.43	(0.11)	***	***	0.09

Impact was measured for the period of 2003–2006 as: a) numbers of publications, b) numbers of citations to those publications by 2012, c) the number of citations received by the most highly cited paper, and d) the number of very highly cited papers. Relationships were determined for three disciplines: Integrative Animal Biology (Bio), Inorganic and organic chemistry (Chem) and Ecology & Evolution (Ecol). In two cases, a break point (B.P.) was estimated visually from LOWESS plots, and the slope estimated using only the points higher than the B.P. We tested slopes for significant difference from 0 (p_b = 0_) and from 1.0 (p_b = 1_). 0.05<p<0.10 #; p<0.01 **; p<0.001; otherwise, p>0.10.

Further, impact is generally a decelerating function of funding. For all disciplines and measures of impact examined, the slope of the log-log relationship is ≤1.0 (Table 1). With b<1, impact per dollar is negatively related to funding (Figure S1b in File S1). Therefore, if maximizing the total impact of the entire pool of grantees is the goal, then the “few big” strategy would be less effective than the “many small” strategy. This conclusion remains true even after eliminating researchers below the threshold (break-point).

Nor do high impact articles reliably flow from large funding. Consider two researchers with average-sized grants, versus one researcher with twice the average funding. Because impact increases with funding (Figures 1, 2, 3), the best article of the more highly-funded researcher is expected to attract 58% more citations than the best article of a single average-funded researcher. However, the relationship between funding and high-impact publications is highly variable (Figures 1, 2, 3). If two researchers at a given funding level are drawn at random, it is likely that one will have much higher impact publications than the other. It can be shown (Appendix S1 in File S1) that, given the observed values of *b* and the variance around the lines in Figures 1, 2, 3, the best article of one rich researcher received, on average, 14% *fewer* citations than the best article from any random pair of researchers, each of whom received only half as much funding (Appendix S1 in File S1)! Further, two small grants yielded 20% more high-impact articles than one large grant. In other words, if high-impact articles are the goal, then spreading grants thinly is more likely to produce them than is concentrating the money in few researchers' hands.

Researchers who held funding from multiple granting councils did not have greater impact than researchers with only NSERC funding. Analyses of covariance of any impact measure as a function of NSERC funding showed no significant difference (p>0.05) between grantees who held (typically much larger) CIHR grants in addition to their NSERC grant, versus those who did not. Nor did holders of FQRNT or CFI funding have higher impact than researchers without that additional funding.

We have no information on researchers' other sources of funding. However, other funding would only change our conclusions if funding success from sources not included in this study were *inversely* related to funding success from NSERC. The opposite seems more likely: researchers who compete most successfully for NSERC funds probably also compete most successfully for funds from other sources. Thus, our study probably underestimates the diminishing returns of high funding. A clear implication for the Canadian granting councils is that it is not productive to allow researchers to receive funding from more than one council. This conclusion is consistent with the observation made by Campbell et al. [12] that researchers funded by the U.S. National Cancer Institute (NCI) were no more productive than researchers funded by the National Cancer Institute of Canada (NCIC), despite the fact that the former received much more funding than the latter.

**Figure 4:**
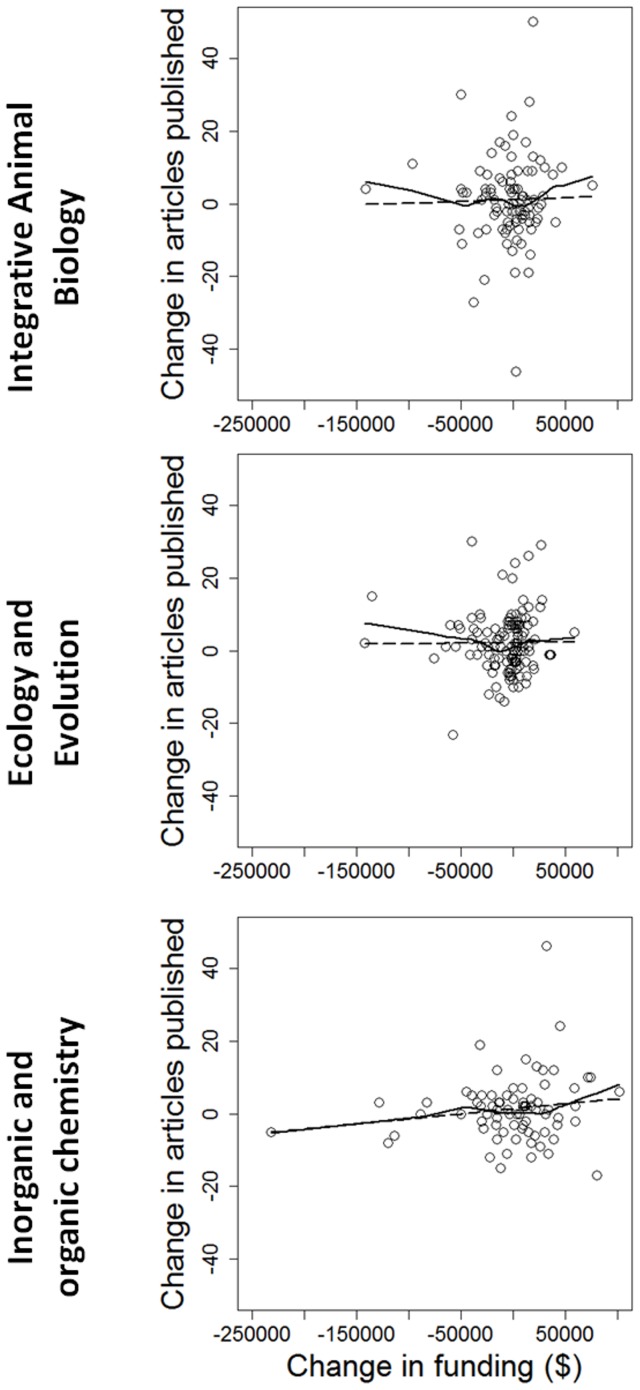
For individual researchers in three disciplines, change in the number of papers published in 2007–2011, in comparison to 2003–2006, expressed as a function of the change in grant funding from the Natural Sciences and Engineering Research Council of Canada from 2006–2010, in comparison to 2002–2005. The solid lines represent LOWESS fits to the data, while the dotted lines represent linear regressions. In all cases, there was no significant relationship.

**Figure 5:**
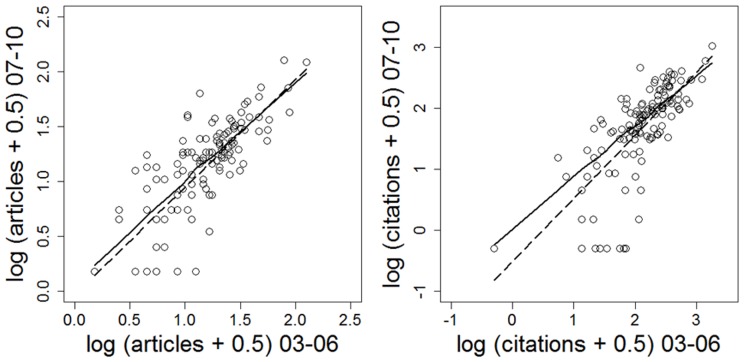
Two measures of scientific productivity measured in 2003–2006 and 2007–2010 for researchers in Integrative Animal Biology. Very similar results were observed for the two other disciplines studied here.

We also find no evidence that granting agencies (or perhaps their grant review panels) can pick (or steer) future productive scientists (in contrast to the conclusion of Campbell et al [12]). Grantees whose funding increased in 2006–2009, relative to 2002–2005, did not show increased scientific impact, on average (Figure 4). Similarly, Berg [14] found that reviewer scores of grant applications submitted to the U.S. National Institutes of Health did not correlate strongly with subsequent productivity. Rather, impact in 2007–2010 was most strongly correlated with impact in 2003–2006 (Figure 5), irrespective of change in funding.

In general, the impact-funding relationships we observed were weak (0.03<R^2^ <0.28). Some of the variance in our results probably comes from methodological limitations. Some papers deriving from the funding period may not have been captured in our impact measures (because they were published later). Some variation in grant sizes is attributable to the cost of the proposed research. This is likely to be relatively small in the case of NSERC grants. The NSERC Peer Review Manual 2012–2013 says that, “It is expected that the majority of applications will be deemed to have normal costs of research.”

Further errors in our bibliometric measures of impact may come from a tendency to cite papers by well-known authors, rather than obscure authors who might deserve more credit (the “Matthew effect” [15]). Further, our study ignored aspects of scientific impact such as books, patents, training of students, etc. However, Dorsey et al [16] found similarly weak relationships between funding of biomedical research and new drug approvals, presumably another measure of impact.

Evidence-based public policy [17] would require evidence that policies yield their intended outcome. Our results are inconsistent with the hypothesis that concentrating research funds on “elite” researchers in the name of “excellence” increases total impact of the scientific community. Quite the opposite: impact per dollar remains constant or decreases with grant size. Highly cited studies are no more likely to result from large grants than from spreading the same funds among multiple researchers.

We postulate that scientific impact, as measured by bibliometric indices, is generally only weakly money-limited (although other important impacts of grant support such as training of students probably do scale closely with funding). Nobel Prize winning research may be highly funded, or not funded at all [18]. At present, we can only speculate about why impact varies so greatly among researchers: perhaps differences in training, in career stage, in other responsibilities (teaching and administrative), in institutional priorities, or perhaps differences in something ineffable, akin to talent. Whatever drives impact, it largely persists through time in individual researchers (Figure 5).

In the absence of statistical evidence in favor of the few-big model, we suggest that there are many other advantages to the many-small model. Each grantee represents an experiment in scientific impact. If the variation of scientific impact among researchers is treated as stochastic, then larger numbers of grantee-experiments will increase the probability of high impact research. The “few big” approach is risky in that it reduces the number of experiments.

A second clear advantage of the many-small model is that at least moderate grant funding serves to keep scientists, and the students around them, active in research. Funding more scientists increases the diversity of fields of research, and the range of opportunities available to students. Greater scientific diversity, like greater genetic diversity, increases the probability that some researcher (like some genetic mutant) will possess characteristics that will flourish in an unpredictable future. It may be relevant that Gordon & Poulin [19] suggest that, given the cost of grant review, it could be more economical simply to give a baseline grant to every qualified researcher (but cf. [20], [21]. Our results suggest that the consequences for scientific impact would not be bad.

Finally, the most unique characteristic of universities is, arguably, their interface between research and teaching [22]. Our results suggest that impact is maximized by funding research as broadly as possible in university communities. This “many small” approach increases the teaching-research interface, and it increases total productivity. We do not deny the impact of some mega-projects such as the Human Genome Project, or the ENCODE project. However, we agree with Alberts, and we feel that the data support his contention that, “Ensuring a successful future for the biological sciences will require restraint in the growth of large centers and -omics-like projects, so as to provide more financial support for the critical work of innovative small laboratories striving to understand the wonderful complexity of living systems.” [23]. To know whether this strategy, or any other (e.g. the model of the Howard Hughes Medical Institute [24]) would actually improve scientific impact requires “turning the scientific method on ourselves” [25]: run the experiment of setting up competing funding systems and see which works best.

## Supporting Information

File S1 Figure S1, Assume that the research impact of individual researchers (*I*), measured in this case as number of publications, varies as an exponential function of grant size (*F*): *I  = aF^b^*, where *a* and *b* are empirical constants. Assume further that any researcher with $10,000 of grant funding produces a single publication. If 0<b<1, impact increases as a decelerating function of funding (panel a). Consequently, researchers with larger grants produce fewer publications per grant dollar (panel b). If b>1, then impact is an accelerating function of funding (panel a) and researchers with large grants produce more publications per dollar than researchers with small grants (panel b). Consequently, if a granting agency has a fixed amount of money to invest (say, one million dollars), then the total impact of all researchers will be greater by spreading the money thinly if 0<b<1 (panel c). In contrast, total impact will be greater by concentrating the funding in the hands of few researchers if b>1. In this study, we find that, for four different measures of scientific impact, the observed value of b is 0≤b≤1. Figure S2, Example of improvement of assumptions typically observed among tested models when untransformed data (panel A) were log transformed (panel B). Residuals vs. fitted and Scale-Location plots both support an improvement on homogeneity of variance between raw and transformed data. Normal Q-Q plots support also that the log transformation improves normality of residual. Residuals vs. Leverage plots support that there are no outliers. This example was made using the data from the Ecology and Evolution committee (n = 139), relating the number of articles published to the amount of NSERC funding received.
(DOCX)
