# Supplementary material for: Big Science vs. Little Science: How Scientific Impact Scales with Funding
Source: PLoS One. 2013 Jun 19;8(6):e65263. doi: 10.1371/journal.pone.0065263 (PMC3686789; doi:10.1371/journal.pone.0065263)
Supplement: File S1 — Figure S1, Assume that the research impact of individual researchers (I), measured in this case as number of publications, varies as an exponential function of grant size (F): I = aFb, where a and b are empirical constants. Assume further that any researcher with $10,000 of grant funding produces a single publication. If 0<b<1, impact increases as a decelerating function of funding (panel a). Consequently, researchers with larger grants produce fewer publications per grant dollar (panel b). If b>1, then impact is an accelerating function of funding (panel a) and researchers with large grants produce more publications per dollar than researchers with small grants (panel b). Consequently, if a granting agency has a fixed amount of money to invest (say, one million dollars), then the total impact of all researchers will be greater by spreading the money thinly if 0<b<1 (panel c). In contrast, total impact will be greater by concentrating the funding in the hands of few researchers if b>1. In this study, we find that, for four different measures of scientific impact, the observed value of b is 0≤b≤1. Figure S2, Example of improvement of assumptions typically observed among tested models when untransformed data (panel A) were log transformed (panel B). Residuals vs. fitted and Scale-Location plots both support an improvement on homogeneity of variance between raw and transformed data. Normal Q-Q plots support also that the log transformation improves normality of residual. Residuals vs. Leverage plots support that there are no outliers. This example was made using the data from the Ecology and Evolution committee (n = 139), relating the number of articles published to the amount of NSERC funding received. (DOCX) [file pone.0065263.s001.docx]

Supporting information

a

b

c

Figure S1 Assume that the research impact of individual researchers (*I*), measured in this case as number of publications, varies as an exponential function of grant size (*F*): *I =aF^b^*, where *a* and *b* are empirical constants. Assume further that any researcher with $10,000 of grant funding produces a single publication. If 0<b<1, impact increases as a decelerating function of funding (panel a). Consequently, researchers with larger grants produce fewer publications per grant dollar (panel b). If b>1, then impact is an accelerating function of funding (panel a) and researchers with large grants produce more publications per dollar than researchers with small grants (panel b). Consequently, if a granting agency has a fixed amount of money to invest (say, one million dollars), then the total impact of all researchers will be greater by spreading the money thinly if 0<b<1 (panel c). In contrast, total impact will be greater by concentrating the funding in the hands of few researchers if b>1. In this study, we find that, for four different measures of scientific impact, the observed value of b is 0≤b≤1.


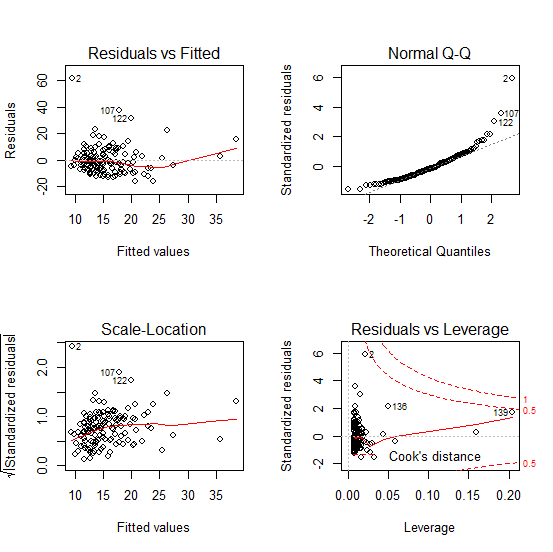


A


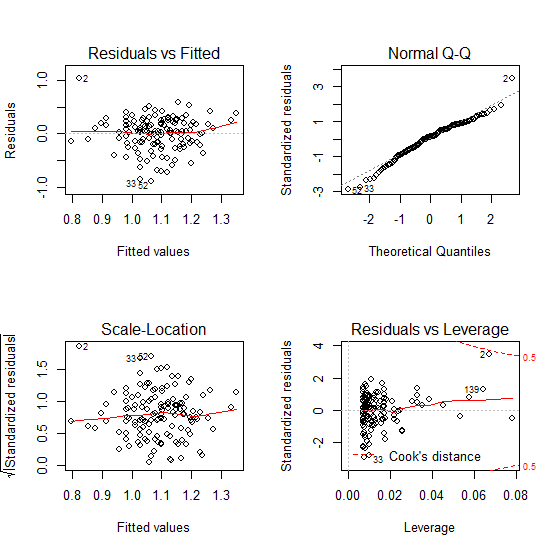


B

Figure S2 Example of improvement of assumptions typically observed among tested models when untransformed data (panel A) were log transformed (panel B). Residuals vs. fitted and Scale-Location plots both support an improvement on homogeneity of variance between raw and transformed data. Normal Q-Q plots support also that the log transformation improves normality of residual. Residuals vs. Leverage plots support that there are no outliers. This example was made using the data from the Ecology and Evolution committee (n=139), relating the number of articles published to the amount of NSERC funding received.

Appendix S1

What is the expected number of citations to the best cited paper (“highest”), or the number of highly cited papers (“excellent”) of :

- (A) One researcher with $2X of grants
- (B) Two researchers, each with $X of grants?

What is the ratio of B/A?

Let *I*= the measure of productivity or impact. We have found that:

*log_10_(I+0.5) =a +b log_10_(X) + ε*

where *a*=intercept, *b* =slope, and *ε* is normally distributed error with mean=0 and variance= *s^2^*. The regression equation thus gives the expected impact for a given level of funding.

Consider the expected impact in two independent draws from this distribution. Let F represent the (normal) probability distribution of ε, and f its probability density function. Drawing two samples from the distribution, the probability that the maximum is less than Y is the probability that both observations are less than Y. Since the two draws are independent, this equals (F(Y))^2^. So, the distribution function of the maximum of two draws is G(Y)= (F(Y))^2^, and its density function is g(Y)=2F(Y)g(Y).

It can be shown with some calculus that, when *ε~N(0,s^2^)*, the expectation of the maximum of ε in two draws is s/(π^0.5^). Thus, if the smaller grants are both X=$160,000 per four years (an average NSERC grant size), then the expected impact of one researcher who has $320,000 is:

A = 10^[a +b log_10_(2X)]-0.5

And the expected impact of the better of two researchers, each of whom has $160,000 is:

B = 10^[a + b log_10_(X) + s/ π^0.5^]-0.5

Given the values of *a, b* and *s* observed in Fig. 1, one can calculate:

| Committee |  | a | b | s | A | B | B/A |
| --- | --- | --- | --- | --- | --- | --- | --- |
| Animal Bio | highest | -1.3009 | 0.5692 | 0.3756 | 67.52 | 74.18 | 1.0986 |
|  | excellent | -1.2552 | 0.02228 | 0.3164 | <0 | <0 |  |
| Chemistry | highest | -2.527 | 0.7883 | 0.5194 | 64.47 | 73.37 | 1.138 |
|  | excellent | -1.2618 | 0.217 | 0.3161 | 0.36 | 0.61 | 1.7139 |
| Ecology | highest | -1.2892 | 0.584 | 0.5006 | 83.80 | 107.26 | 1.28 |
|  | excellent | -2.2195 | 0.4272 | 0.3242 | 0.86 | 1.04 | 1.211 |

where “highest” denotes the number of citations to the most highly cited paper, and “excellent” denotes the number of highly cited papers. The values for *a, b* and *s* all come from the regressions shown Fig. 1. *A* represents the expected value of these measures of impact for a researcher with a grant twice the average amount.  *B* represents the expected impact of the more productive of the two researchers, each of whom has an average-sized grant. The ratio *B/A* >1 shows that, for all committees and for both measures impact, two average funded researchers are more likely to produce high-impact work than one researcher with twice as much money.
